# Supplementary material for: Cutaneous Allodynia of the Withers in Cattle: An Experimental In Vivo Neuroanatomical Preliminary Investigation of the Dichotomizing Sensory Neurons Projecting into the Reticulum and Skin of the Withers—A Case Study on Two Calves
Source: Animals (Basel). 2025 Jun 6;15(12):1689. doi: 10.3390/ani15121689 (PMC12189413; doi:10.3390/ani15121689)
Supplement: Supplementary file 1 [file animals-15-01689-s001.zip › animals-3612801-supplementary.pdf]

Supplementary Materials:

Figure S1 - The injection sites of the fluorescent retrograde tracers Fast Blue and Diamidino Yellow.

Figure S2 - The deposit of the Diamidino Yellow tracer in the skin.

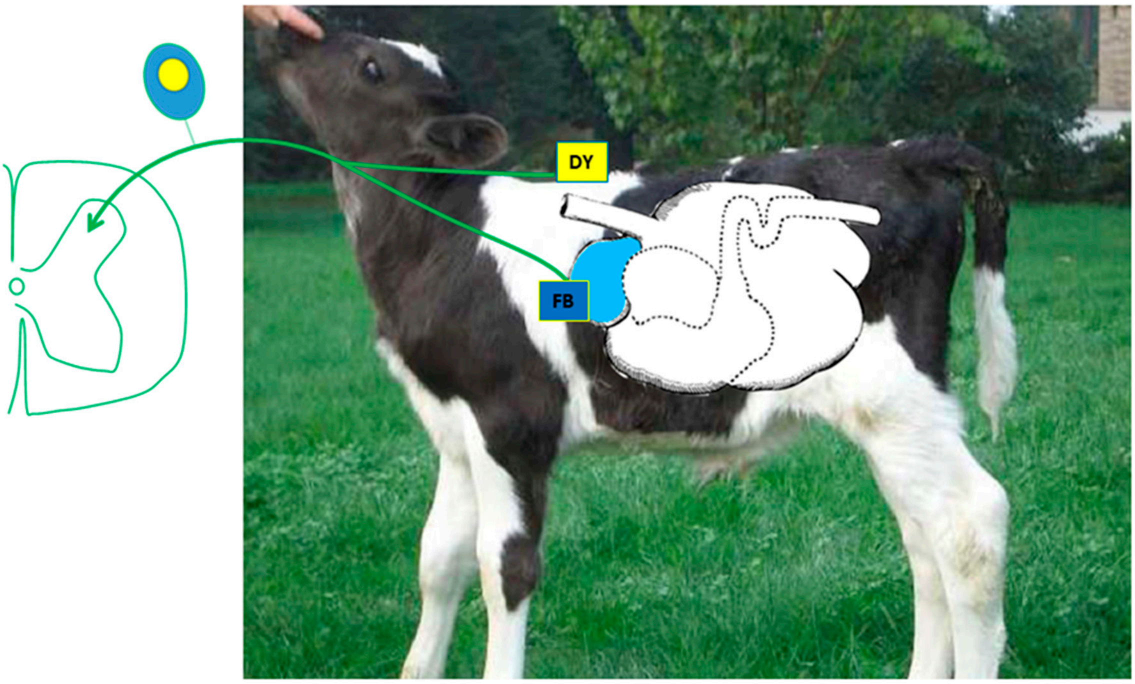

Supplementary figure S1. The image illustrates the injection sites of the two fluorescent retrograde tracers used in the study. The Fast Blue tracer, which labels the cytoplasm of the primary sensory neurons, was injected into the cranial face of the *reticulum*, while the Diamidino Yellow tracer, which labels the neuronal nuclei, was injected into the skin of the withers.

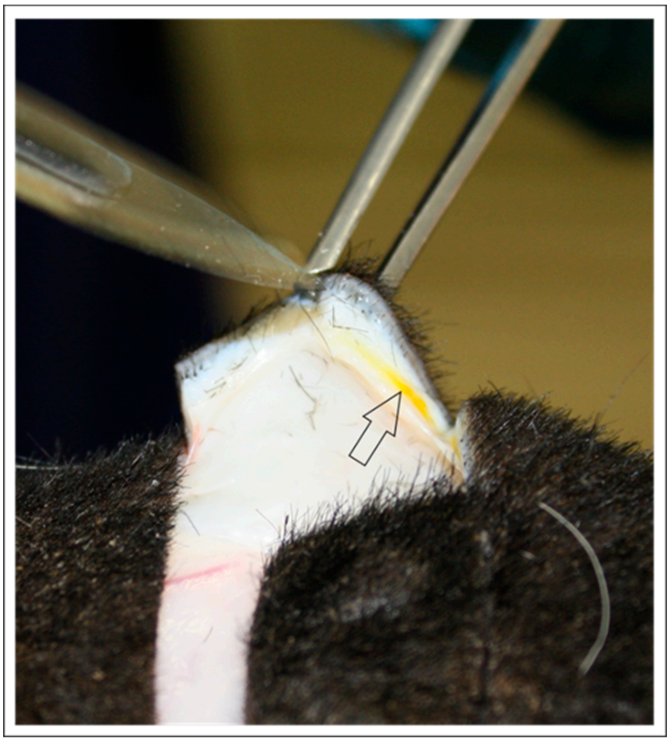

Supplementary figure S2. The photograph shows the skin incision of the weaned calf (calf #1) in which the deposit of the Diamidino Yellow tracer is visible, one month after its injection in the skin of the withers.
